# Selective isolation of ammonia-oxidizing bacteria from autotrophic nitrifying granules by applying cell-sorting and sub-culturing of microcolonies

**DOI:** 10.3389/fmicb.2015.01159

**Published:** 2015-10-16

**Authors:** Hirotsugu Fujitani, Asami Kumagai, Norisuke Ushiki, Kengo Momiuchi, Satoshi Tsuneda

**Affiliations:** Department of Life Science and Medical Bioscience, Waseda UniversityTokyo, Japan

**Keywords:** ammonia oxidation, cell sorter, microcolony, nitrification, *Nitrosomonas*, uncultured

## Abstract

Nitrification is a key process in the biogeochemical nitrogen cycle and biological wastewater treatment that consists of two stepwise reactions, ammonia oxidation by ammonia-oxidizing bacteria (AOB) or archaea followed by nitrite oxidation by nitrite-oxidizing bacteria. One of the representatives of the AOB group is *Nitrosomonas mobilis* species. Although a few pure strains of this species have been isolated so far, approaches to their preservation in pure culture have not been established. Here, we report isolation of novel members of the *N. mobilis* species from autotrophic nitrifying granules used for ammonia-rich wastewater treatment. We developed an isolation method focusing on microcolonies formation of nitrifying bacteria. Two kinds of distinctive light scattering signatures in a cell-sorting system enabled to separate microcolonies from single cells and heterogeneous aggregates within granule samples. Inoculation of a pure microcolony into 96-well microtiter plates led to successful sub-culturing and increased probability of isolation. Obtained strain Ms1 is cultivated in the liquid culture with relatively high ammonia or nitrite concentration, not extremely slow growing. Considering environmental clones that were closely related to *N. mobilis* and detected in various environments, the availability of this novel strain would facilitate to reveal this member’s ecophysiology in a variety of habitats.

## Introduction

Nitrification is an important process in the biogeochemical nitrogen cycle and biological wastewater treatment. This process consists of two stepwise reactions, ammonia oxidation by ammonia-oxidizing bacteria (AOB) or archaea and nitrite oxidation by nitrite-oxidizing bacteria (NOB). Traditionally, biochemical and physiological properties of AOB have been characterized based on studies using representative pure strains. However, most nitrifying microorganisms are difficult to cultivate and isolate by conventional culture-based methods and cultivation-independent molecular methods have revealed high diversity and predominance of members of this group of microorganisms that have yet to be cultured.

*Nitrosomonas* and *Nitrosospira* belonging to *Betaproteo bacteria* are the most widely distributed AOB in the environment (Purkhold et al., 2000). This group comprises phylogenetically distinct clusters, and members of each cluster have unique physiological characteristics. The physiological, biochemical, and genetic characteristics of pure strains isolated from each cluster have been investigated by many researchers. Cluster 6A is represented by the *Nitrosomonas oligotropha* lineage, which consists of members that are relatively ammonia-sensitive and found in freshwater, wastewater, estuaries, and terrestrial systems (Koops et al., 1991; Suwa et al., 1994; Coci et al., 2005, 2008; Fierer et al., 2009). Members of cluster 6B, including *N. marina* and *N. aestuarii*, are tolerant from higher salt concentrations and commonly isolated from marine systems (Koops et al., 1991). Cluster 7 includes *N. europaea* (Winogradsky, 1892), *N. eutropha* (Koops et al., 1991) and other pure strains that can tolerate high ammonia concentrations. Members of this cluster have been isolated from a variety of environments.

*Nitrosomonas mobilis* species also belongs to cluster 7. Since the first identification of this species by Koops et al. (1976), a few pure strains of this species have been isolated from nitrifying biofilms (Juretschko et al., 1998; Purkhold et al., 2000). However, approaches to preserve pure cultures of these strains have not yet been established (Campbell et al., 2011). Although AOB obtain ATP by oxidizing ammonia to nitrite, they produce nitrite and decrease pH, leading to a decrease in AOB activity (Claros et al., 2013). In batch cultures, AOB are sensitive to ammonia and increasing nitrite due to high ammonia concentrations, while AOB could grow in co-culture with NOB consuming nitrite produced by AOB. When AOB are isolated as pure culture, the AOB are doomed to be exposed elevated nitrite concentration. This inevitable condition often leads to the difficulty of long preservation and recovery (Bollmann et al., 2011).

Conventionally, AOB were isolated using different isolation techniques such as limiting dilutions, serious dilutions, and plates solidified with gellan gum or agar. Although these techniques are easy to handle and useful, the conventional methods could decrease the possibility of isolation of novel strains. Therefore, development of a new method is required in any age. Focusing on the formation of microcolonies of nitrifying bacteria, we previously developed an isolation method and obtained novel strains of bacteria in the phylum *Nitrospirae*, which is the most dominant NOB in the wastewater treatment process (Ushiki et al., 2013; Fujitani et al., 2014). Selective inoculation of a pure microcolony, not a single cell as a growth unit by a cell-sorting system led to successful sub-culturing and increased probability of isolation of uncultured NOB. We believed that this method would be applicable to other nitrifying bacteria, such as AOB capable of microcolony formation. Here, we report novel isolates belonging to the *N. mobilis* species from autotrophic nitrifying granules enriched in an ammonia-rich wastewater treatment system (Tsuneda et al., 2003; Matsumoto et al., 2010). It was demonstrated that this isolation method could have general potential to separate AOB based on the microcolony formation and to lead to isolation of uncultured AOB.

## Materials and Methods

### Sample Source

Seed sludge was sampled from the nitrification stage of a municipal wastewater treatment plant in Japan. Nitrifying granules were produced by an aerobic upflow fluidized bed reactor according to a previously described method (Tsuneda et al., 2003) and microbial population dynamics and community structure during the formation of nitrifying granules were analyzed (Matsumoto et al., 2010). The community was composed of the phyla *Proteobacteria*, *Actinobacteria*, *Chloroflexi*, *Bacteroidetes*. The influent ammonia concentration in the bioreactor was about 300 mg-N L^-1^, and the eﬄuent ammonia concentration was maintained below 30 mg-N L^-1^ for 3 years.

### Sorting of Microcolonies from Nitrifying Granules

**FIGURE 1:**
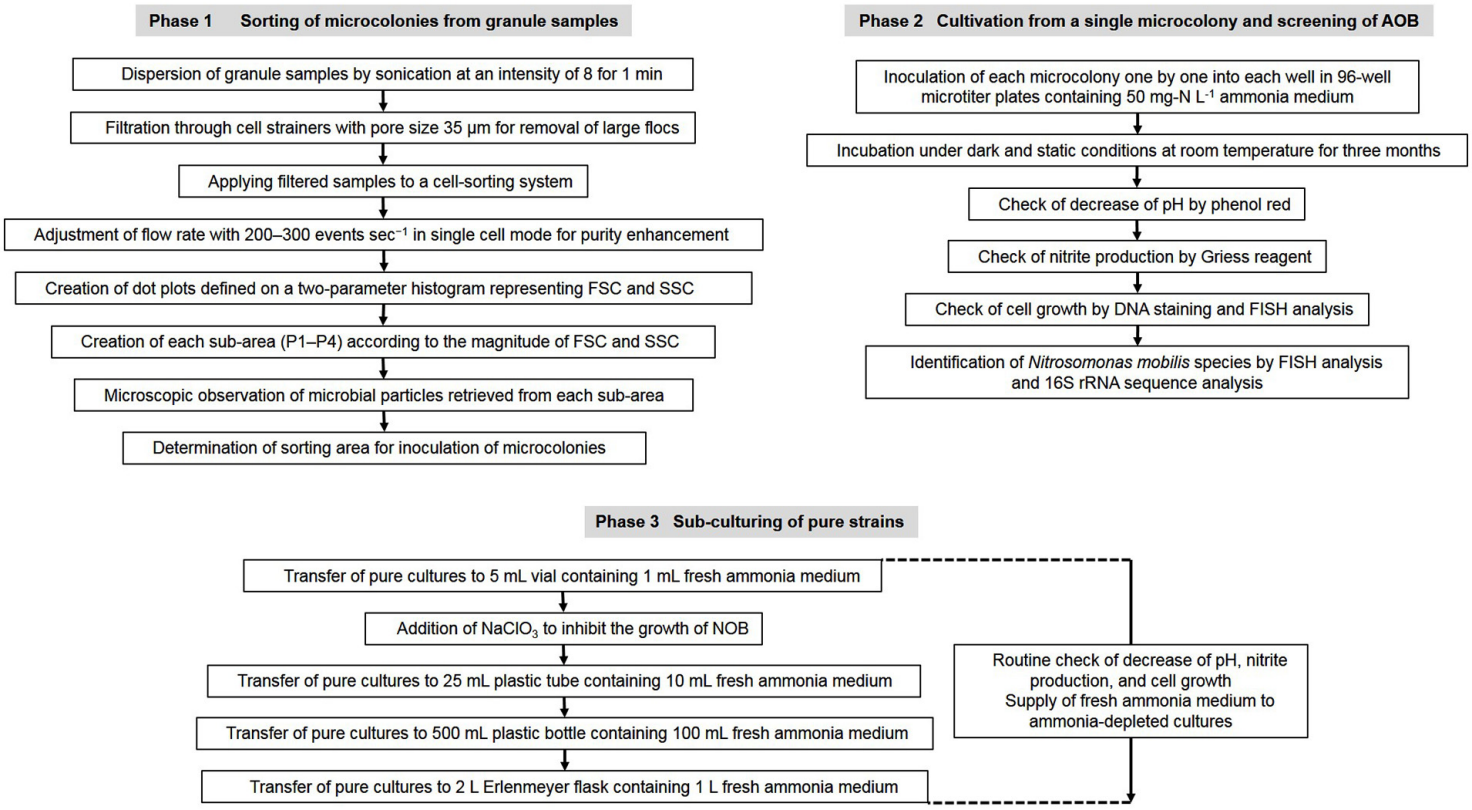
**Flow chart of the experimental procedure to isolate *Nitrosomonas mobilis* species from nitrifying granules and transfer the pure cultures**.

Nitrifying bacterial microcolonies were separated from multi-species clumps using a cell sorter as described in our previous research (Ushiki et al., 2013; Fujitani et al., 2014). Briefly, nitrifying granules were dispersed by sonication at intensity of 8 for 1 min (Sonifier II model 150, Branson, Danbury, CT, USA). The dispersed samples were filtered through cell strainers (5 mL polystyrene round-bottom tubes with cell-strainer caps, pore size 35 μm, Falcon, Co., Ltd., Nagoya, Japan) to remove large flocs and then applied to a cell sorter. Flow rate was adjusted to approximately 200–300 events sec^-1^ in single cell mode for purity enhancement. The dot plot area defined on a two-parameter histogram, which consists of forward scatter (FSC) and side scatter (SSC), was identified and at least 10,000 particles were analyzed for each histogram. Finally, fractions separated from each region (P1–P4) of the dot plot area were mounted onto glass slides (at least 500 particles per slide) and analyzed by fluorescent *in situ* hybridization (FISH; **Figure 1**).

### FISH and DNA Staining

All *in situ* hybridizations were conducted in hybridization buffer at 46°C for 2.5 h according to the standard protocol (Amann et al., 1990). The applied oligonucleotide probes were NmV (specific for *N. mobilis* lineage: 5′- TCC TCA GAG ACT ACG CGG -3′) (Pommerening-Röser et al., 1996) and Nso190 (specific for *Nitrosomonas* genus in *Betaproteobacteria* class: 5′- CGA TCC CCT GCT TTT CTC C -3′) (Mobarry et al., 1996). Oligonucleotides were synthesized and fluorescently labeled with a hydrophilic sulfoindocyanine dye (Cy3) or fluorescein isothiocyanate at the 5′ end (Fasmac Co. Ltd., Atsugi, Japan). SYTOX Green nucleic acid stain (Life Technologies, Carlsbad, CA, USA) was applied as a universal cellular stain. Stained cells were detected and recorded using a confocal laser scanning microscope (IX71, Olympus, Tokyo, Japan) and fluorescence microscopy (Axioskop 2 plus, Carl Zeiss, Oberkochen, Germany).

### Cultivation and Isolation

A fraction containing mostly *N. mobilis* microcolonies was identified. Single microcolonies of *N. mobilis* separated by a cell sorter were incubated for 3 month in 96-well microtiter plates containing 50 mg-N L^-1^ ammonia medium composed of NH_4_Cl (228 mg L^-1^), NaCl (116 mg L^-1^), MgSO_4_⋅7H_2_O (40.0 mg L^-1^), CaCl_2_⋅2 H_2_O (73.0 mg L^-1^), KCl (38.0 mg L^-1^), KH_2_PO_4_ (34.0 mg L^-1^), FeCl_2_⋅6H_2_O (2 mg L^-1^), MnCl_2_⋅4H_2_O (100 μg L^-1^), CoCl_2_⋅6H_2_O (24 μg L^-1^), NiCl_2_⋅6H_2_O (24 μg L^-1^), CuCl_2_⋅2H_2_O (17 μg L^-1^), ZnCl_2_ (68 μg L^-1^), Na_2_MoO_4_ (24 μg L^-1^), and H_3_BO_3_ (62 μg L^-1^). The final pH was adjusted to 7.8 and phenol red was used as a pH indicator. Cultivation was conducted under dark and static conditions at room temperature (23°C). Cell growth was assessed by FISH analysis and fluorescence microscopy, while nitrite production was confirmed using Griess reagent (Shinn, 1941). Pure bacterial strains grown in the 96-well microtiter plates were identified by analyses of their 16S rRNA and *amoA* gene sequences (**Figure 1**).

### DNA Extraction and PCR Amplification

DNA was extracted from pure strains using the ISOIL extraction kit (Nippon Gene, Tokyo, Japan) according to the manufacturer’s instructions. The 16S rRNA gene fragment (ca. 1,500 bp) of the total DNA was amplified using the 27f/1492r primer set (27f: 5′-AGAGTTTGATCATGGCT-3′ and 1492r: 5′-TACGGTTACCTTGTTACGACTT-3′). The following thermal profiles were used for the 16S rRNA gene amplification: an initial denaturing step of 95°C for 2 min, followed by 30 cycles of denaturation at 94°C for 1 min, annealing at 55°C for 1 min, and elongation at 72°C for 1 min and then final extension at 72°C for 2 min. The PCR reaction mixtures (50 μL) were composed of 10× PCR buffer, 0.2 μM of each primer, 2.5 mM of dNTPs, and Ex Taq DNA polymerase (TakaraBio, Otsu, Japan).

The *amoA* gene fragment (ca. 490 bp) of the total DNA was amplified using the bacterial primers amoA1F/amoA2R (Rotthauwe et al., 1997). The following thermal profiles were used for *amoA* gene amplification: initial denaturation at 94°C for 3 min, followed by 35 cycles of denaturation at 94°C for 30 s, annealing at 52°C for 30 s, and elongation at 72°C for 30 s. The final extension step was conducted at 72°C for 10 min.

### Phylogenetic Analysis

Alignment editing and phylogenetic analyses were performed using ChromasPro version 1.4.1 (Technelysium Pty, Tewantin, Australia) and the MEGA4.0 software (Tamura et al., 2007). The bacterial 16S rRNA gene sequences were compared with those available in the DNA Data Bank of Japan (DDBJ). Genetic distance was calculated using a p-distance model of nucleic acid substitution.

### Subculture of Novel Strains

Novel strains unambiguously identified as *N. mobilis* were transferred to the same medium used for isolation prior to use in the physiological experiments. Ammonia-depleted cultures were supplied with fresh ammonia medium and pure cultures were maintained in 2 L Erlenmeyer flasks containing 1 L of medium. During cultivation, NaClO_3_ was added to medium to inhibit the growth of NOB. According to previous researches, 10 mM NaClO_3_ inhibits the oxidation of nitrite to nitrate by *Nitrobacter* (Lees and Simpson, 1957; Belser and Mays, 1980) but does not affect the oxidation of ammonia to nitrite by *N. europaea* (Belser and Mays, 1980). Based on the report, the same concentration of NaClO_3_ was added in this study. Ammonia consumption and nitrite production were monitored using a water test kit (Kyoritsu Chemical Check Lab, Tokyo, Japan), Griess reagent and ion chromatography (IC 2001, Tosoh, Tokyo, Japan). Cell growth was checked by microscopic observation (**Figure 1**).

### Purity Test and Preservation

The growth of heterotrophic contaminants was tested using both agar-plate and liquid media containing 200-fold dilution of the given DNB formula (peptone 1 mg L^-1^, meat extract 0.6 mg L^-1^) and DR2A (polypeptone 2.5 mg L^-1^, casamino acid 2.5 mg L^-1^, sodium pyruvate 1.5 mg L^-1^, soluble starch 2.5 mg L^-1^, yeast extract 2.5 mg L^-1^, KH_2_PO_4_ 2.5 mg L^-1^, MgSO_4_7H_2_O 0.25 mg L^-1^). The temperature was maintained at 23°C, and cultivation was conducted under dark and static conditions for 2–6 months. The contamination was checked by FISH analysis with NIT3, a probe specific for *Nitrobacter* (5′- CCT GTG CTC CAT GCT CCG-3′) (Wagner et al., 1996), and by PCR with primers 27f/1050r specific for the genus of *Nitrobacter* (5′-CACCTGTGCTCCATGCTCCG-3′) (Freitag et al., 2005). To establish a protocol for preservation of pure strains, some strains were preserved under low temperature (4°C, –20°C, and –80°C) in dimethyl sulfoxide (DMSO) medium or in inorganic medium containing ammonia with the same composition as that used for cultivation and isolation.

### Chemical Analyses

Ammonia concentration was checked by colorimetric analysis with indophenol. Nitrite and nitrate concentrations were determined quantitatively by ion chromatography (IC 2001, Tosoh, Tokyo, Japan). The samples for the ammonia oxidation activity test were filtered through 0.20 μm cellulose acetate membrane filters (Advantec, Tokyo, Japan).

### Electron Microscopy

Electron microscopic analysis was conducted at Hanaichi Ultrastructure Research Institute, Okazaki, Japan, as previously described (Fujitani et al., 2014).

## Results

### Sorting of Microcolonies from Nitrifying Granules

**FIGURE 2:**
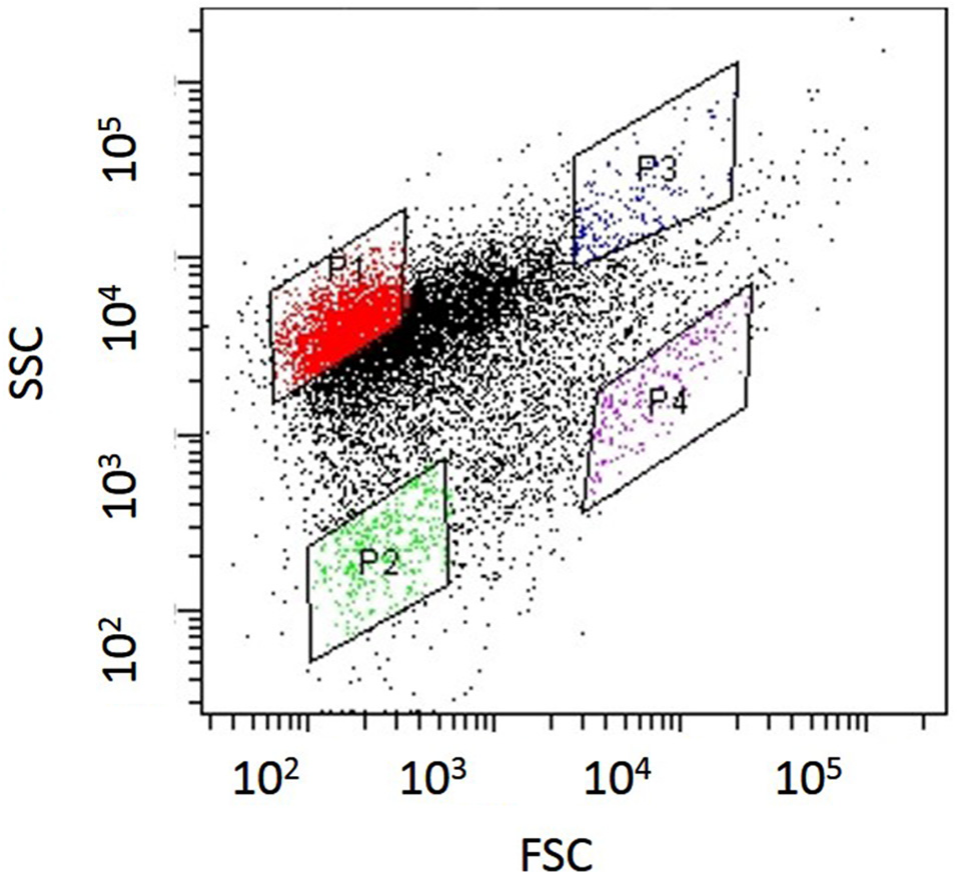
**Dot plots obtained from nitrifying granule samples via sonication and cell-sorting techniques**. The identified dot plot area was divided into four sub-areas (P1–P4).

**FIGURE 3:**
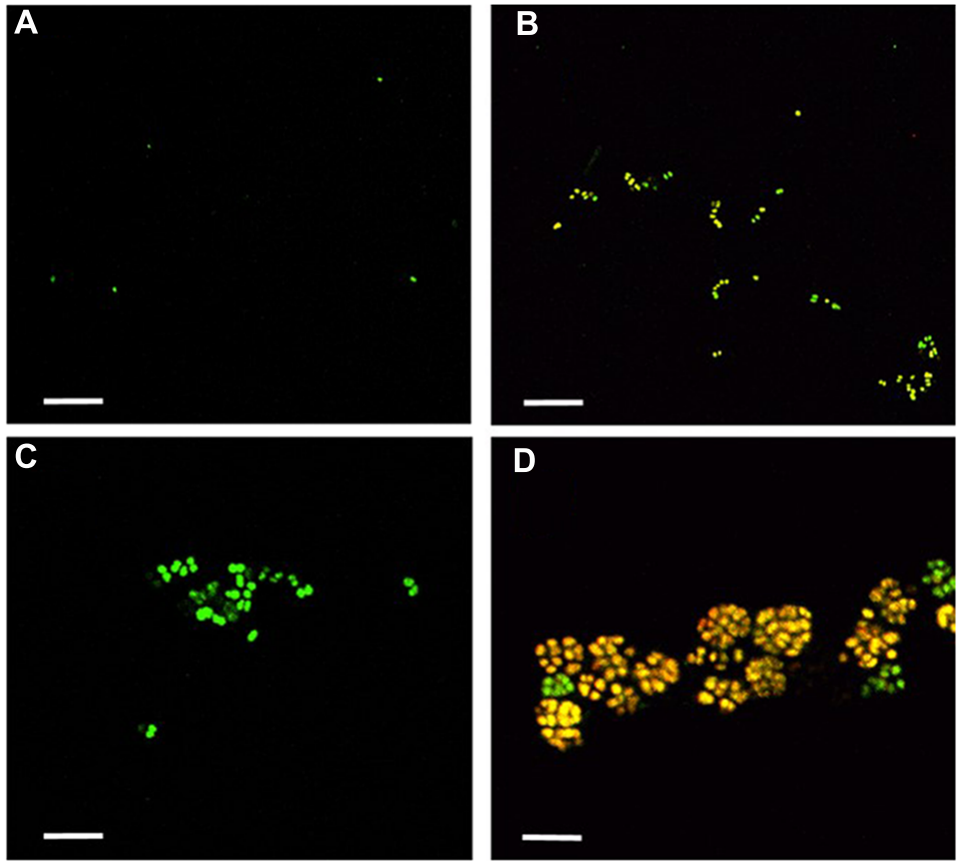
**Fractions sorted from each area (P1–P4) were observed by confocal laser scanning microscopy**. Yellow cells show the NmV-stained *N. mobilis* species. Green cells are SYTOX green-stained other microorganisms. Scale bars indicate 5 μm. **(A)** P1, **(B)** P2, **(C)** P3, and **(D)** P4.

*Nitrosomonas mobilis* is the dominant AOB in nitrifying granules in an aerobic upflow fluidized bed reactor. The ratio of *N. mobilis* cells to the total microorganisms was 30–40%, while any other AOB were not detected by FISH analysis. After the nitrifying granule samples were dispersed by ultrasonic treatment and applied to a cell sorter, the areas of the generated dot plots were determined (**Figure 2**) according to our previous studies (Ushiki et al., 2013; Fujitani et al., 2014). Briefly, the dot plot area was divided into four sub-areas (P1–P4) consisting of different magnitudes of FSC representing size of particles and SSC representing complexity of particles. Fractions separated from each sub-area were then subjected to microscopic observation. As expected, planktonic cells and small microcolonies tended to occupy the P1 and P2 areas, indicating a low FSC fraction (**Figures 3A,B**). The P3 area included larger and more complex multi-species aggregates (**Figure 3C**), whereas the P4 area in the dot plot contained most of the pure *N. mobilis* microcolonies (**Figure 3D**).

Before application to a cell sorter, the population of planktonic single cells or multi-species aggregates was much larger than that of microcolonies. The ratio of *N. mobilis* microcolonies to the number of total events always remained extremely low (below 1%) in the original sample. However, the ratio of *N. mobilis* cells dramatically increased in the P4 area resulting from cell-sorting. FISH analysis revealed that the ratio of *N. mobilis* microcolonies to the number of total events was 62.3% when using the NmV probe specific to *N. mobilis* and 59.7% in response to Nso190 probe specific to *Nitrosomonas*. Based on microbial cell counting, the ratio of *N. mobilis* cells increased to 99% of individual microbial cells under both probes. Therefore, the P4 area showing high FSC and low SSC values was the appropriate area to purify *N. mobilis* from other bacteria. The portions remaining in the P4 area were aggregates, other bacterial microcolonies, single cells, debris, or vacant counts.

### Screening of Positive Wells and Pure Cultivation

*Nitrosomonas mobilis* microcolonies sorted from the P4 area were individually inoculated into 96-well microtiter plates containing medium with a relatively low ammonia concentration (50 mg-N L^-1^) and cultivated under dark and static conditions over 3 months. After cultivation, microscopic observation elucidated the growth of pure single-species cells, contaminants, and no cells in each well. Single *N. mobilis* microcolonies were inoculated into 576 wells in total and 180 wells showed a decrease in pH. Nitrite production was subsequently identified in 24 of 180 wells by Griess reagent. FISH analysis and microscopic observation confirmed the growth of *N. mobilis* in 12 of 24 wells. Finally, the samples in these 12 positive wells were subjected to PCR amplification and sequence analysis. The remaining 12 wells showing nitrite production except for *N. mobilis* were not identified due to the existence of other bacteria and contamination with heterotrophic bacteria.

### Phylogenetic Analysis

**FIGURE 4:**
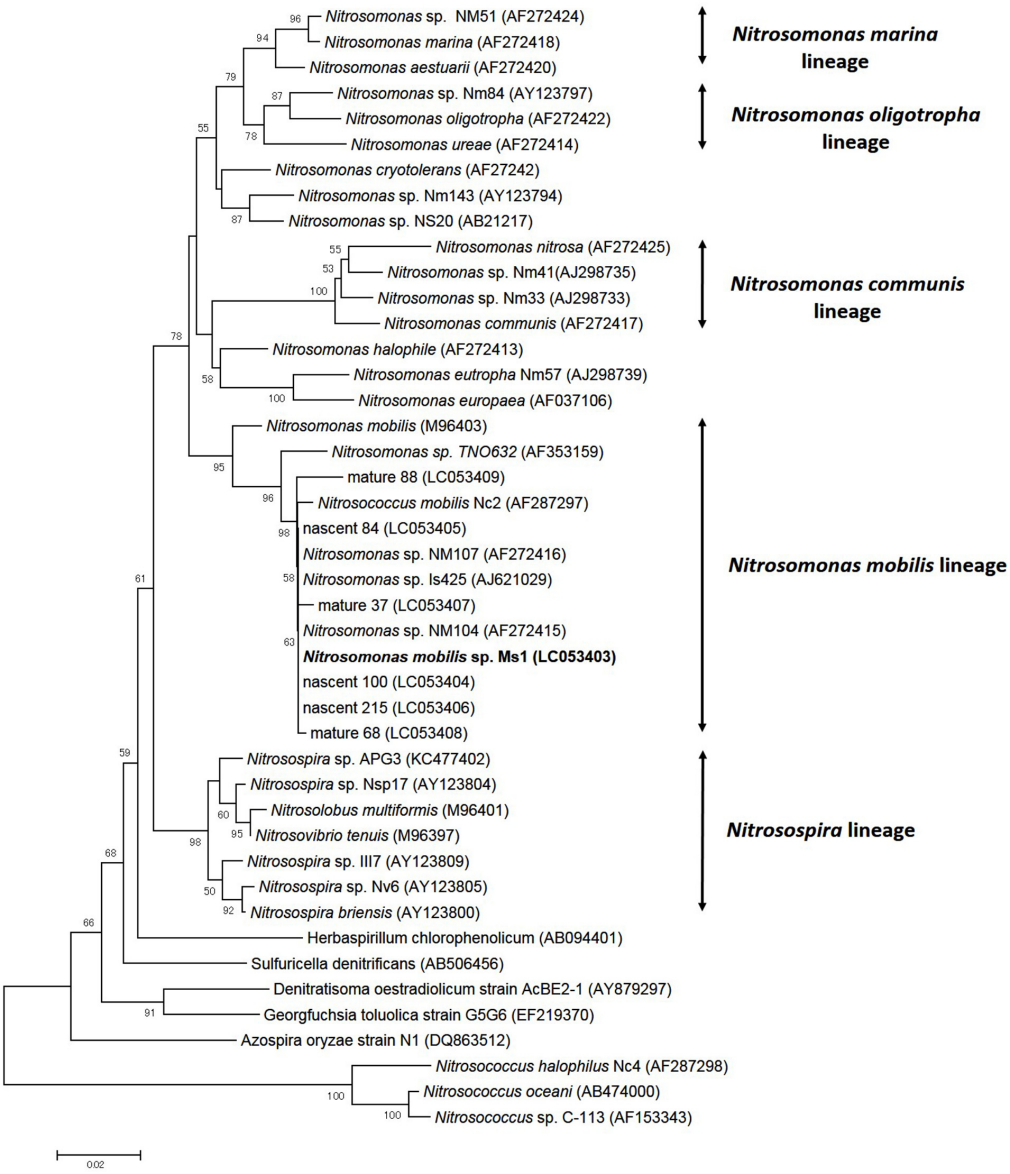
**Phylogenetic analysis showing the affiliation of isolates in this study**. The phylogenetic tree is based on 16S rRNA gene sequences of selected *Betaproteobacteria*. The tree was constructed using the neighbor-joining algorithm. Numbers at the branch nodes are bootstrap values. The scale bar corresponds to the 2% estimated sequence divergence.

**FIGURE 5:**
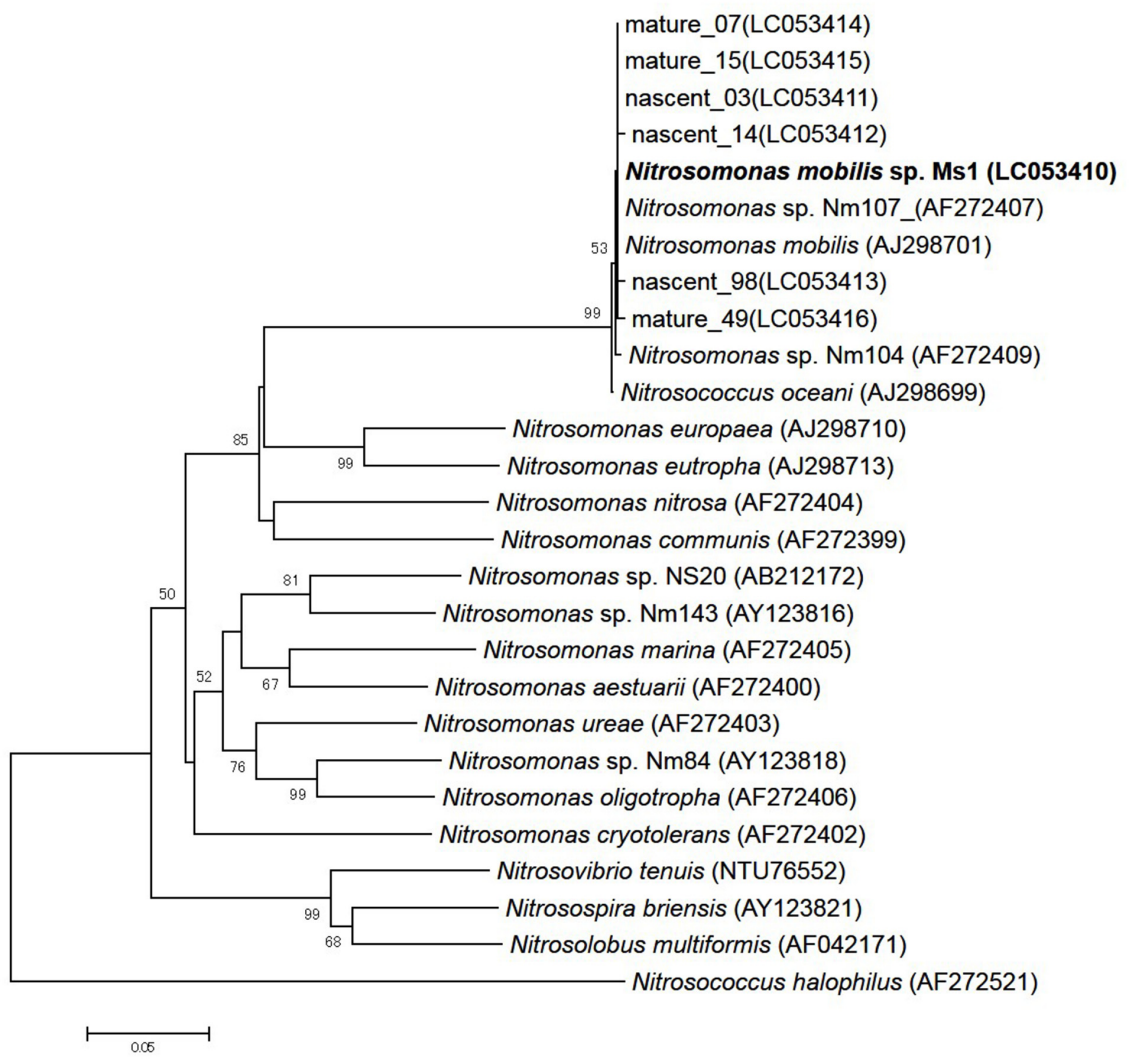
**Phylogenetic analysis based on bacterial *amoA* gene sequences of selected *Nitrosomonas* class**. The tree was constructed using the neighbor-joining algorithm. Numbers at the branch nodes are bootstrap values. The scale bar corresponds to the 5% estimated sequence divergence.

To identify pure strains in 12 wells, 16S rRNA gene sequences and *amoA* gene sequences were analyzed. Although four samples could not be identified because of low DNA level, the remaining eight were attributed to the *N. mobilis* lineage in the class of *Betaproteobacteria*. The sequence similarity within the eight samples was 99–100% and one representative strain was named *N. mobilis* sp. Ms1. Similarities between strain Ms1 and other known AOB were compared at the 16S rRNA gene level and *amoA* gene level (**Table 1**). At the 16S rRNA gene level, 100, 100, and 99.6% homology with *Nitrosomonas* sp. NM 107, *Nitrosomonas* sp. NM 104, and *Nitrosococcus mobilis* Nc 2 was observed, respectively (**Figure 4**; **Table 1**). At the *amoA* gene level, high similarities (99–100%) were observed within the *N. mobilis* lineage on both DNA and amino acid level. When compared with clones obtained from nitrifying granules, similarities with nascent clones and mature clones were nearly 100% (**Figure 5**; **Table 1**). Overall, these results demonstrated that strain Ms1 obtained in this study was unambiguously affiliated with *N. mobilis* from autotrophic nitrifying granules.

**Table 1:** Similarity of *Nitrosomonas mobilis* sp. Ms1 with other AOB pure strains or clones retrieved from nitrifying granules.

Species/clones	*amoA* (amino acid) (%)	*amoA* (DNA) (%)	16S rRNA (DNA) (%)	Accession number (16S)	Accession number (*amoA*)
*Nitrosomonas* sp. Nml07	100	100	100	AF272416	AF272407
*Nitrosomonas* sp. Nml04	100	99.7	100	AF272415	AF272409
*Nitrosococcus mobilis* Nc2	–	–	99.6	AF287297	–
*Nitrosococcus oceani*	100	99.7	83.6	AB474000	AJ298699
Nascent 03	100	100	–	–	LC053411
Mature 49	100	99.7	–	–	LC053416
*Nitrosomonas nitrosa*	87.3	80	91.4	AF272425	AF272404
*Nitrosomonas europaea*	87.3	75.4	93.5	AF037106	AJ298710
*Nitrosomonas eutropha*	86.4	80	93.3	AJ298739	AJ298713
*Nitrosomonas communis*	81.4	75.7	92.8	AF272417	AF272399
*Nitrosomonas aestuarii*	80.5	72.9	94.4	AF272420	AF272400
*Nitrosomonas cryotolerans*	78.8	72.3	95.3	AF27242	AF272402
*Nitrosomonas oligotropha*	78.8	72	94.1	AF272422	AF272406
*Nitrosomonas ureae*	78	73.4	93.3	AF272414	AF272403
*Nitrosospira briensis*	78	66.1	94.4	AY123800	AY123821
*Nitrosolobus multiformis*	78	66.1	93.3	M96401	AF042171
*Nitrosomonas marina*	77.1	66.8	94.2	AF272418	AF272405
*Nitrosovibrio tenuis*	75.4	66.7	93.7	M96397	NTU76552
*Nitrosococcus halophilus*	42.4	49.7	83.1	AF272413	AF272521

*In previous studies, granules with an average diameter of 126 and 270 μm were defined as nascent granules and mature granules, respectively (Matsumoto et al., 2010). The description of nascent and mature in a species/clones column means clones obtained from original granules*.

### Purity Check and Preservation

The purity of the culture was carefully inspected throughout the experiment by (i) FISH microscopic observation, (ii) transferring of cultures to several types of organic culture medium and (iii) PCR. FISH revealed no contaminants in the cultures. The inner portions of the microcolonies assembled in pure culture were further inspected by confocal laser scanning microscopy to confirm the purity and each microcolony was found to consist solely of *N. mobilis* cells. The purity of the cultures was also repeatedly checked by successive transfers to both agar-plate and liquid media containing 200-fold diluted Nutrient Broth (DNB) (BD, Franklin Lakes, NJ, USA) and R2A (DR2A) to detect other microbes capable of growing in oligotrophic conditions and no growth was observed in any of the test samples. To investigate whether pure cultures were contaminated with NOB co-existing in the original granule sample, FISH and PCR using the *Nitrobacter*-specific probes and primers, respectively, confirmed the absence of *Nitrobacter*.

### Morphology

**FIGURE 6:**
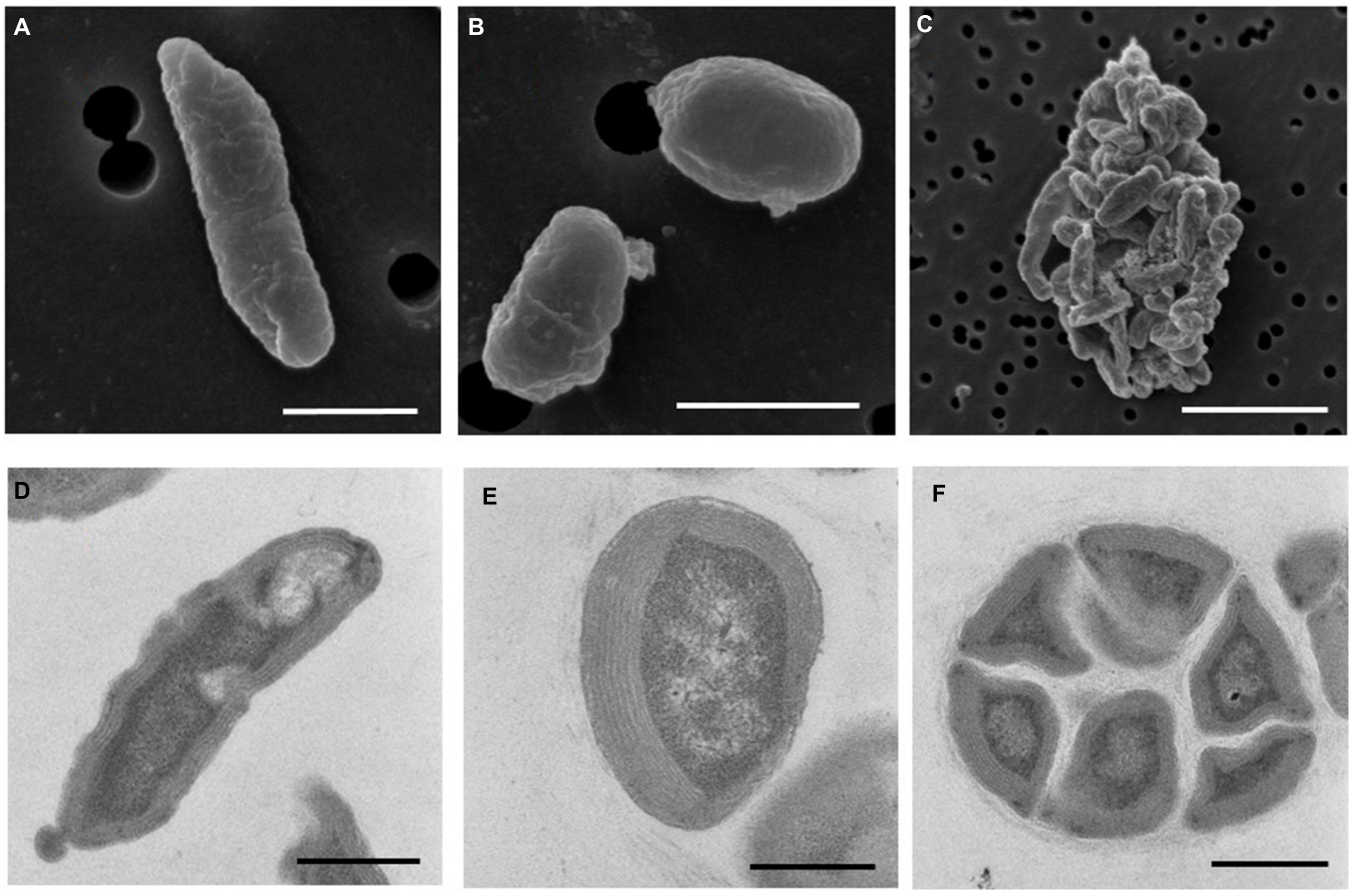
**Morphology of *N. mobilis* sp. Ms1 isolated from nitrifying granules**. Scanning electron microscopic images of **(A)** a rod-shaped cell (scale bar = 1 μm), **(B)** a spherical cell (scale bar = 1 μm), and **(C)** a microcolony (scale bar = 5 μm). Transmission electron microscopic images of **(D)** a rod-shaped cell (scale bar = 0.5 μm), **(E)** a spherical cell (scale bar = 0.5 μm), and **(F)** a microcolony (scale bar = 1 μm).

Electron microscopy revealed that strain Ms1 has unique morphological characteristics. Scanning electron microscopy (SEM) revealed that the width and length of the rod-shaped cells ranged from 0.3 to 0.6 μm and 0.8 to 3 μm, respectively (**Figure 6A**). Additionally the diameter of spherical cells was about 0.8–1.0 μm (**Figure 6B**). A microcolony consisted of approximately 20–50 cells and each cell was tightly connected (**Figure 6C**). Transmission electron microscopy (TEM) of ultrathin sections of the rod-shaped cells or spherical cells revealed that each cell was surrounded with multi-layers of cytoplasmic membrane, with a thickness of 200–500 nm (**Figures 6D,E**). Individual cells within a microcolony were packed densely (**Figure 6F**).

## Discussion

In this study, novel pure strains affiliated with *N. mobilis* were isolated from autotrophic nitrifying granules. Our group previously developed a novel isolation and cultivation method for uncultured NOB. This method focused on morphological features of microorganisms and enabled selective separation of microcolonies of NOB. Indeed, novel strains belonging to the phylum *Nitrospirae*, which forms dominant NOB species in activated sludge in wastewater treatment plants, were isolated and characterized (Ushiki et al., 2013; Fujitani et al., 2014). In the next challenge, we applied this technique to AOB. Conventionally, pure strains of *N. mobilis* were isolated by forming colonies on agar plates (Koops et al., 1976; Juretschko et al., 1998; Purkhold et al., 2000). In this study, use of liquid medium instead of solid medium enabled pure cultivation. Previous research reported that agar as a gelling reagent often prevents cell growth (Tamaki et al., 2009) and furan-2-carboxylic acid in agar inhibited colony formation of some types of bacteria (Hara et al., 2012). Although it was tested whether strain Ms1 isolated in this study grow on agar plates with the same medium composition as liquid medium, the cell growth was not confirmed. Therefore, this novel method was effective for microorganisms capable of colony formation in liquid medium.

### Prevention of Contaminants

The most important issue in isolation of AOB is the removal of contaminants such as heterotrophic bacteria and NOB. Although separating and sorting of *N. mobilis* microcolonies are not laborious, very few cells of *Nitrobacter* attaching to the microcolonies grew throughout the long cultivation procedure. Because NOB acquires energy by oxidizing nitrite produced by AOB, *Nitrobacter* attaches firmly to *N. mobilis* in nitrifying granules. Indeed, RNA sequencing revealed that *N. europaea* showed increased growth activity in co-culture with *Nitrobacter* (Perez et al., 2015); therefore, *N. mobilis* might also gain some benefit from co-existence with this partner. The addition of NaClO_3_ as an inhibitor of nitrite oxidation effectively prevented the growth of *Nitrobacter*. Moreover a previous study reported that *N. mobilis* might interact with *Nitrospira* in biofilms in activated sludge samples (Juretschko et al., 1998). However, *Nitrospira* was not detected in the procedure of pure cultivation in this study.

### Culturability and Subculture

Inoculating microcolonies, instead of single cells might trigger the growth of *N. mobilis* cells. This is because microcolony formation creates ‘high local cell density’ conditions. Indeed, Batchelor et al. (1997) reported that the growth activity of *N. europaea* increased at a higher cell density, probably because such activity is controlled by quorum sensing mechanisms. Although it is not known if *N. mobilis* is involved in cell to cell communication, inoculation with a high cell density of microcolonies could initiate the growth.

Even though *N. mobilis* microcolonies were inoculated into 576 wells, sufficient cell multiplication for DNA sequence analysis was only observed in eight wells, indicating that the cultivability was 1.4%. Our previous research also demonstrated that the cultivability of NOB was only 1–3% (Ushiki et al., 2013; Fujitani et al., 2014). Therefore, the cultivability showed nearly the same values in any different types of nitrifiers. It has been reported that most microorganisms in the environment are dormant (e.g., viable but not cultivable; Lennon and Jones, 2011); therefore, the relationship between low cultivability and dormancy of *N. mobilis* should be investigated. This low cultivability has other reasons. In the sorting process, sonication to disperse flocs and laser of a cell sorter might damage cells. During cultivation, toxic substances eluted from plastic microtiter plates, pH decrease and nitrite production in medium could affect the growth of cells.

The doubling time of strain Ms1 was 12–24 h, which agreed with the results reported by Koops et al. (1976). This value was not very different from those of other AOB pure strains. Since AOB are generally difficult to transfer and preserve as pure cultures, slow growth rate of AOB might contribute to the difficulty. However, strain Ms1 is cultivated in liquid medium with relatively high ammonia or nitrite concentrations, which would enable sub-culturing to investigate the physiological characteristics of this strain.

Conventionally, *N. europaea* has been investigated genetically and biochemically in many studies because of ease in handling. However, *N. europaea* is not widely distributed in all environments in which AOB flourish. Environmental clones closely related to *N. mobilis* were detected in activated sludge in wastewater treatment plants, recirculating aquaculture systems, and wetlands (Limpiyakorn et al., 2005; Wang et al., 2012; Kumar et al., 2013). Therefore, the availability of this novel *N. mobilis* strain would facilitate to reveal this species’ ecophysiology in a variety of habitats.

## Conclusion

In this study, novel AOB pure strains belonging to *N. mobilis* species were isolated from autotrophic nitrifying granules. Utilization of a cell-sorting system with both light scattering signatures (FSC and SSC) enabled separation of AOB microcolonies with high efficiency and without specific labeling, resulting in pure culture of *N. mobilis*. Therefore, the isolation method conducted in this study could have a general potential to isolate uncultured AOB capable of microcolony formation.

## Conflict of Interest Statement

The authors declare that the research was conducted in the absence of any commercial or financial relationships that could be construed as a potential conflict of interest.
